# Nudge strategies to improve healthcare providers’ implementation of evidence-based guidelines, policies and practices: a systematic review of trials included within Cochrane systematic reviews

**DOI:** 10.1186/s13012-020-01011-0

**Published:** 2020-07-01

**Authors:** Sze Lin Yoong, Alix Hall, Fiona Stacey, Alice Grady, Rachel Sutherland, Rebecca Wyse, Amy Anderson, Nicole Nathan, Luke Wolfenden

**Affiliations:** 1 Hunter New England Population Health, University of Newcastle, Locked Bag 10, Wallsend, New South Wales 2287 Australia; 2 School of Medicine and Public Health, The University of Newcastle, Callaghan, New South Wales 2308 Australia; 3 Hunter Medical Research Institute, Newcastle, New South Wales 2300 Australia; 4 Priority Research Centre for Health Behaviour, The University of Newcastle, Callaghan, New South Wales 2308 Australia

**Keywords:** Implementation intervention, Nudge, Healthcare provider, Guidelines, Review

## Abstract

**Background:**

Nudge interventions are those that seek to modify the social and physical environment to enhance capacity for subconscious behaviours that align with the intrinsic values of an individual, without actively restricting options. This study sought to describe the application and effects of nudge strategies on clinician implementation of health-related guidelines, policies and practices within studies included in relevant Cochrane systematic reviews.

**Methods:**

As there is varied terminology used to describe nudge, this study examined studies within relevant systematic reviews. A two-stage screening process was undertaken where, firstly, all systematic reviews published in the Cochrane Library between 2016 and 2018 were screened to identify reviews that included quantitative studies to improve implementation of guidelines among healthcare providers. Secondly, individual studies within relevant systematic reviews were included if they were (i) randomised controlled trials (RCTs), (ii) included a nudge strategy in at least one intervention arm, and (iii) explicitly aimed to improve clinician implementation behaviour. We categorised nudge strategies into priming, salience and affect, default, incentives, commitment and ego, and norms and messenger based on the Mindspace framework.

**Synthesis:**

The number and percentage of trials using each nudge strategy was calculated. Due to substantial heterogeneity, we did not undertake a meta-analysis. Instead, we calculated within-study point estimates and 95% confidence intervals, and used a vote-counting approach to explore effects.

**Results:**

Seven reviews including 42 trials reporting on 57 outcomes were included. The most common nudge strategy was priming (69%), then norms and messenger (40%). Of the 57 outcomes, 86% had an effect on clinician behaviour in the hypothesised direction, and 53% of those were statistically significant. For continuous outcomes, the median effect size was 0.39 (0.22, 0.45), while for dichotomous outcomes the median Odds Ratio was 1.62 (1.13, 2.76).

**Conclusions:**

This review of 42 RCTs included in Cochrane systematic reviews found that the impact of nudge strategies on clinician behaviour was at least comparable to other interventions targeting implementation of evidence-based guidelines. While uncertainty remains, the review provides justification for ongoing investigation of the evaluation and application of nudge interventions to support provider behaviour change.

**Trial registration:**

This review was not prospectively registered.

Contributions to the literature

- This review describes the application and potential impact of nudge strategies on clinician implementation behaviour for trials included in Cochrane systematic reviews, and seeks to add to the field by highlighting strategies that may be typically overlooked in design of implementation interventions.
- This review suggests that nudge strategies, even when not part of a multicomponent intervention, could be useful in improving clinician behaviour.
- There is a need to better describe and classify nudge strategy in order to fully understand its impact on implementation in the healthcare setting.

## Introduction

Evidence-based guidelines are developed specifically to improve the effectiveness of healthcare providers professional practice, reduce the risk of unintended adverse effects to the community and are fundamental tools for the translation of research into practice [1]. Research studies, however, consistently document poor implementation of evidence-based clinical guidelines [2]. Consequently, a large volume of research examining ‘how’ to best support healthcare providers to implement evidence-based clinical guidelines now exists. The Cochrane Effective Practice and Organisation of Care (EPOC) group have published over 100 systematic reviews which describe interventions designed to improve professional practice and the delivery of effective health services by implementing evidence-based clinical guidelines and practices [3]. A systematic review of reviews in primary care alone found 91 reviews which evaluated strategies such as audit and feedback, educational meetings and other practitioner targeted interventions, to improve clinical guideline adherence [4]. The intervention approaches and interpretations synthesised within these reviews have largely assumed that practitioners behave in a rational manner. As such, these interventions have actively targeted rational constructs such as attitudes, intentions and self-efficacy in an attempt to change behaviour.

However, recent behavioural research suggests that in many instances, an individual’s behaviour and decision-making is not always perfectly rational [5]. Dual process models propose that individual decision-making and behaviour results from the interaction between two cognitive processes operating in parallel, one reflective, and the other impulsive or automatic [6]. In the provision of diabetes care, for example, studies have reported that ‘automatic’ decision-making processes operate alongside, and may mediate rational processes in influencing clinician provision of evidence-based care [7]. Additionally, a recent meta-analysis of nine studies assessing the association between ‘habit’ (which is an indicator of the automatic decision-making process) and clinician behaviour reported a moderate correlation (*r* = 0.33) [5, 8]. Such research suggests that interventions to change clinician behaviour need to move beyond strategies that focus purely on rational cognitive pathways, towards considering the context within which individual’s act, which are influenced by automatic pathways.

Nudge strategies have been suggested as one way to influence habitual or automatic behaviour, by targeting the subconscious routines and biases that are present in human decision-making and behaviour [9]. ‘Nudging’ was first coined in 2008 and is defined as a ‘function of any attempt at influencing people’s judgement, choice or behaviour in a predictable way, that is made possible because of cognitive boundaries, biases, routines and habits in individual and social decision-making’ (p158) [9]. These boundaries, biases and routines act as barriers for people to perform rationally consistent with their internal values. As such, nudge strategies work by targeting these boundaries, biases and habits by altering the underlying ‘choice architecture’, the social and physical environment in which the decision is made [9]. Specifically, ‘choice architecture’ involves the design of different ways in which choices can be presented to individuals. This can include influencing the range of choices (e.g. increasing number, types of choices), considering the manner in which the attributes are described (e.g. labelling, priming) and altering the way in which an object is presented (e.g. as a default, placement, presentation, sizing) [10]. This enhances the capacity for subconscious behaviours that aligns with the intrinsic values of an individual, without actively restricting options [11].

Nudge strategies are considered highly appealing from a policy and public health perspective as they are low cost and typically do not require ongoing resources to sustain. These interventions have been applied in public health policy to change behaviour and support healthier lifestyle choices. For example, many governments have applied nudges in the form of altered defaults, switching from opt in to opt out systems to increase organ donation rates. The United Kingdom (UK) Institute for Government and Behavioral Insights Team developed the Mindspace framework as a way to support the application of nudge strategies in public policy [12]. This framework describes the nine types of interventions (Mindspace: Messenger, Incentives, Norms, Defaults, Salience, Priming, Affect, Commitments, Ego) that are considered to have the most robust effects on the automatic system. Mindspace is underpinned by core principals of behavioural economics and aligns closely with other seminal lists describing nudge and choice architecture techniques [12, 13]. As such, this framework provides a structured way to support administrators, policy makers and researchers with selecting and applying nudge interventions to influence behaviour [12].

The potential impact of nudge strategies on clinician implementation behaviour has only recently started to be formally evaluated. A randomised controlled trial by Meeker found that a simple intervention encouraging practitioners to display a public poster stating their commitment to reduce inaccurate antibiotic prescription in waiting rooms, significantly reduced inappropriate prescribing rates relative to the control group (− 19.7% [95% confidence interval: − 5.8% to − 33.4]; *p* = 0.02) [14]. The UK Behavioural Insights team undertook a randomised 2 × 2 factorial trial examining the impact of providing social norm feedback to high antibiotic-prescribing GPs within their team. The study found that providing information concerning providers’ prescribing rate, compared with other local practices (norm) in the area from England’s chief medical officers (messenger), significantly reduced the rate of antibiotic items dispensed per 1000 population (*p* < 0.0001) [15].

Given the potential of ‘nudge’ strategies to impact on clinicians’ behaviours, efforts to describe the application and potential effect of such strategies on implementation are warranted. An overview of the type of settings and behaviours that have been targeted can highlight opportunities for future empirical research, and inform the design of low-cost implementation strategies.

This review aims to describe the application and effects of nudge strategies on healthcare provider and organisations’ implementation of evidence-based guidelines, policies and practices. The review will use data from randomised controlled trials included within Cochrane systematic reviews

## Methods

This review has been reported in accordance to the PRISMA guidelines [16]. This review was not registered, and a protocol has not been previously published.

### Information sources and search strategy

The nudge terminology was developed in 2008 and has gained popularity in the last decade. Implementation trials testing the impact of nudge strategies, however, may have been performed over many decades. Many interventions that would have been classified as nudge are not published under this term. Given this, conventional electronic searches of bibliographical databases are unlikely to be sufficiently sensitive or specific, and are likely to result in conflated numbers and potentially missed studies. To avoid this, we conducted a targeted and systematic search of studies included within eligible Cochrane systematic reviews. The Cochrane library was chosen as it is internationally recognised for publishing up to date, high-quality and current systematic reviews in healthcare settings, and has a review group dedicated to publication of studies to improve healthcare professional practices [17]. We undertook a two-stage screening process, where systematic reviews were screened for eligibility and following that, trials within eligible reviews identified in the first process assessed for inclusion. Two authors (SLY, FS) screened the titles, abstract and full text of all reviews published by the Cochrane library in the last 2 years (2016–2018) on December 2018. This timeframe was selected as Cochrane authors are encouraged to update their reviews every 2 years.

### Study selection

Following this, full text of all included studies within eligible reviews were screened by at least two authors (SLY, FS, RS). Studies were included if they met all eligibility criteria described below. All disagreements were resolved via a consensus process between the two reviewers and involved a third reviewer (LW) where necessary.

### Eligibility criteria

All studies that examined the impact of a nudge strategy targeting clinician and healthcare organisation’s implementation of health-related guidelines, policies and practices were included if they met the following criteria.

#### Types of studies

Only randomised controlled trials (RCTs) with a parallel control group that compared (i) an intervention that included a nudge strategy to improve the implementation of a healthcare-related guideline, policy and practice in healthcare settings/organisations compared with a non-nudge intervention or usual practice; and (ii) two or more different strategies, which included at least one arm with a nudge strategy, to improve the implementation of a healthcare-related guideline, policy and practice were included. Studies also had to specify the implementation of a health-related policy, guideline and practice as an explicit aim of the study, and as such were likely to be type 2 or type 3 hybrid trials [18].

#### Type of participants

Study participants were clinicians (medical doctors, allied health professionals) providing care in healthcare/clinical settings. Healthcare settings included acute care hospitals; long-term care facilities, such as nursing homes and skilled nursing facilities; physicians’ offices (i.e. primary care); urgent-care centres; outpatient clinics; home healthcare (i.e. care provided at home by professional healthcare providers) and emergency medical services [19].

#### Type of intervention

The intervention had to include at least one nudge strategy. The determination of whether a nudge strategy was present was undertaken by at least three reviewers (SY, FS, AA) for each study. Nudge strategies were defined as those that ‘applied principles from behavioural economics and psychology to alter behaviour in a predictable way without restricting options or significantly changing economic incentives’ (p6) [11]. These strategies were those that targeted the automatic decision-making processes rather than the rational decision-making processes [20]. Strategies were classified using the Mindspace framework (see Table 1) [20]. The Mindspace framework was chosen as it provides a practical checklist for summarising the application of nudge strategies in public health practice. Similar to a previous review [21], strategies were classed as (i) priming, (ii) norms and messenger, (iii) salience and affect, (iv) default, (v) commitment and ego and (vi) incentives [22]. For studies with multiple intervention arms (e.g. multi-arm RCTs, factorial trials or comparative effectiveness trials), only the arm/s that included an intervention with a nudge strategy were included. Multiple intervention arms with nudge strategies were combined as in most instances the intervention arms included the same type of nudge strategy. Where the intervention was multicomponent and included both nudge and non-nudge strategies, this was also included. Trials that included a nudge strategy in the control arm were excluded to allow the impact of the nudge strategies to be assessed relative to no nudge strategy.

**Table 1:** Nudge categories and description applied in the review based on the Mindspace framework

Categories of nudges using the Mindspace framework	Description
Priming	Subconscious cues which might be physical, verbal or sensational and are changed to nudge a particular choice
Salience/affect	Novel, personally relevant vivid examples and explanations that are used to increase attention to a particular choice
Default	A particular choice is ‘preset’, making it the easiest option
Incentive	Incentives to reinforce a positive choice, or penalties to discourage a negative choice. Such incentive however should not be enough to result in economic gains
Commitment/ ego	Making a commitment/ ego or public promise in order to elevate one’s desire to feel good about themselves
Norms and messenger	Using the practices of peers or others to establish a norm. People of status, professional organisations and peer leaders used to communicate with individuals

*Mindspace* Messenger, Incentives, Norms, Default, Salience, Priming, Affect, Commitment, Ego

#### Type of outcomes

We included any subjective or objective measure of implementation outcomes. Similar to previous reviews carried out by the team [23, 24], implementation outcomes were those that described the fidelity or execution of a guideline, policy or practice at an organisational or practitioner level. Such outcomes could be assessed via self-reported surveys, observations or from other routine data sources including electronic medical records. Examples of such outcomes include appropriate prescribing or test ordering in line with guideline recommendations.

Studies were excluded if they were not published in English. There were no other exclusions beyond that specified by the original review the studies were extracted from.

### Data extraction

Relevant information was extracted from the published Cochrane reviews, the primary trial and other associated papers referenced in the primary trial by at least two individuals (FS, RS, JJ, BM) using a standardised data extraction form. This included (1) study information—author name, study design, country, date of publication, type of healthcare provider/organisation, participant/service demographic/socioeconomic characteristics and number of experimental conditions; (2) characteristics of the overall implementation strategy, including the duration, number of contacts and approaches to implementation, and information to allow classification of the intervention strategy into nudge categories according to Table 1 (priming, norms and messenger, salience and affect, commitment and ego, incentives and default nudge); (3) trial primary or summary outcome measures and results, including the data collection method, validity of measures used, effect size (or raw data that allowed the calculation of an effect size) and measures of outcome variability; and (4) risk of bias assessment as published in the Cochrane reviews [25]. Where several implementation outcomes were reported, we extracted only the results and risk of bias assessment for those explicitly described as the primary outcome(s) of the trial, for all follow-up time periods. Where the primary outcome was not specified in the individual trial, we extracted the variable(s) described in the sample size calculation.

### Data analysis

To describe the application of nudge strategies in practice, we calculated the number and percentage of trials using each nudge strategy, according to the Mindspace framework. We also described the application of nudge strategies according to setting and type of outcomes assessed. Where the primary outcomes were clearly identified in the aims or via sample size calculation, we calculated the within-study effect for this outcome. Where there were several primary outcomes, we focused on all implementation outcomes and calculated a pooled effect size for that study, if the outcomes were similar. Where they were different, we calculated the within-study effect for each outcome separately. We also calculated the within-study effects for outcomes reported at multiple time-points for each time-point.

Due to substantial clinical and methodological heterogeneity of included trials, it was not appropriate to conduct a meta-analysis. Instead, we summarised the effect estimates and used vote-counting methods, as outlined in the Cochrane Handbook, for where a meta-analyses was not possible [26]. We calculated within-study point estimates and 95% confidence intervals (CIs). For all studies, we extracted the raw values (mean, standard deviation, median interquartile range (IQR) and range for continuous outcomes; and percentages and frequencies for dichotomous outcomes). We used this data in the estimation of within-study effects. For continuous outcomes, we calculated the effect size as the difference in follow-up scores between intervention and control, except for one study that only reported the change in outcomes for control and intervention. The difference in change scores was used for this study [27]. For dichotomous outcomes, we calculated odds ratio (OR) as the measure of intervention effect. Odds ratios were chosen as it is a relative measure that is less sensitive to differences in baseline values than absolute measures such as risk differences. Additionally, ORs are also not as influenced by the underlying prevalence of the outcome as other relative measures [28]. We calculated within-study effects for all outcomes together with 95% CIs. The direction of a favourable intervention effect varied across studies, with some studies aiming for a reduction, and others aiming for an increase in a behaviour. Where studies aimed for a reduction, we reverse-scored these values. To provide an overview of overall impact, and by nudge strategy, we reported the number of studies and outcomes with an estimated effect in the beneficial direction as well as the percentage of effects favouring the intervention. These results were summarised in harvest plots, which visually demonstrate the directional effects of an intervention strategy and are recommended to help summarise review results when meta-analysis is not appropriate [29]. Finally, we calculated the median standardised mean effect size for continuous outcomes and median OR for dichotomous outcomes and IQR.

Statistical analyses were programmed using SAS v9.4 [30], Stata v13.0 and R [31, 32].

#### Clustered studies

All clustered trials were examined for unit of analysis errors to calculate within-study effects. For cluster randomised trials, the effective sample size was calculated and used for all estimates of effect sizes. This was undertaken to allow for inclusion in the harvest plots so that the potential impact of nudge strategies can be considered in light of the size of the studies. To calculate the effective sample size, the intracluster correlation co-efficient (ICC) derived from the trial (if available) or from another source (for example, the ICC used in the sample size calculation, or the mean ICC calculated from the other included studies) was used, and the design effect calculated using the formula provided in the Cochrane Handbook for Systematic Reviews of Interventions [33].

#### Studies with more than two treatment groups

Procedures described in the Cochrane Handbook for Systematic Reviews of Interventions [33] were followed for trials with more than two intervention or comparison arms that included a nudge strategy. This involved combining multiple intervention arms following the recommended formula set out by the Cochrane Handbook. Only intervention arms that included relevant nudge strategies as part of the intervention package were combined and compared to the control group. Multiple comparison arms were combined, rather than described separately, to help focus this review.

## Results

### Review characteristics

A total of 1730 systematic reviews published in the last 2 years (2016–May 2018) were obtained from the Cochrane library database. Forty-three full-text reviews were included in the full text screen and of those, 36 reviews were excluded for the following reasons: did not examine implementation outcomes (*n* = 33) [34–66], not undertaken in healthcare settings (*n* = 1) [67], not quantitative (*n* = 1) [68], or was a review of previous systematic reviews (*n* = 1) [69].

A total of seven reviews that examined a range of health-related practices including antibiotic prescribing [70], hand hygiene [71], management of obesity [72], management of musculoskeletal conditions [73], uptake of clinical guidelines across health behaviours [74, 75], and provision of mental healthcare [76] were included.

**Fig. 1:**
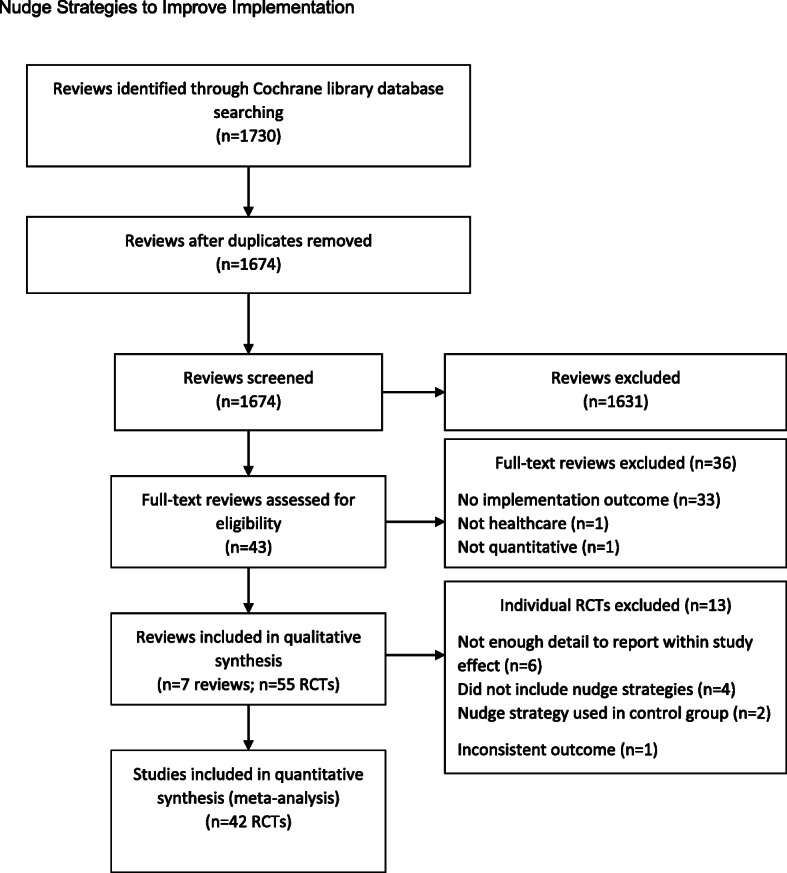
PRISMA flowchart of study selection process

From these seven included reviews, 55 eligible RCTs that met all the inclusion criteria were included. Of these, 13 studies were excluded from the analysis: six did not report sufficient detail for calculation of a within-study effect [77–82], six included a nudge strategy in the control group [83–88], and one reported using an inconsistent outcome (time to event) [89]. Thus, a total of 42 studies, reporting across 57 outcomes, were included in the final analyses. Figure 1 contains a PRISMA flowchart of the study selection process.

### Study characteristics

Table 2 provides an overview of the included studies. Most studies were conducted in the USA (40%; *n* = 17), Canada (21%; *n* = 9) and the UK (14%; *n* = 6). Over half included two experimental arms, including the control (*n* = 26; 62%), 24% included three arms (*n* = 10) and 14% (*n* = 6) included four arms. Over half of the studies were cluster trials (52%, *n* = 22), one was a cross-over RCT [102] and the remainder were simple RCTs. Over half of the studies employed a control group that consisted of usual care or no intervention strategy (62%, *n* = 26). Over a quarter used a minimal intervention (26%, *n* = 11) which included strategies such as provision of guidelines, written recommendations, and introduction of facilities or equipment, and 7% (*n* = 3) used an intervention as part of the control, including education sessions to provide information; reminders and feedback targeting adequate products and facilities, provision of encouragement, monitoring and feedback and support. A further two studies [123, 125] did not provide sufficient detail of the control group.

**Table 2:** Study characteristics of included trials in the review by nudge strategy

Author name, year of publication, study design, country	Setting/healthcare professional	Number of experimental conditions	Guidelines targeted	Control group description	Multicomponent strategy (yes (Y)/no (N) ); Description of intervention	Description of nudge strategy	Implementation outcomes (primary), time-point/s	Data collection method	Effect size—SMD/OR (95% CI) reversed
Priming nudge									
Baer 2015 [90] , cluster RCT, USA	12 primary care practices affiliated with an academic medical centre	2	Obesity management	Usual care	Y; Educational presentation; resources; additional information about the electronic health record & guidelines	Prompts to assess if body mass index is not assessed within the last year, reminders and other resources provided at point of care	Percentage of patients with a documented body mass index in the medical record within 12 months after initial visit	Collected as part of routine medical records (the electronic health records or scheduling systems)	OR 0.91 (0.30,2.75)
Barnett 1983 [91], RCT, USA	One physician group practice	2	None specific	Usual care	N; Physicians were sent reminders that they had deviated from standard care, and also an encounter form to record when next follow-up should occur. Another reminder was sent if follow-up specified by physician was not completed	Automated computer-generated reminder and encounter form to record when next follow-up should be	Adherence to quality assurance recommended programme; follow-up attempted or achieved at 6-12 months and 12-24 months	Self-encoding checklists unique to each specialty are completed by the physician or nurse at the time of the patient encounter and subsequently entered in to the system (usually on the same day) by record room personnel	OR 15.90 (6.32,39.99)
Burack 1996 [92], RCT, USA	Two sites of a health maintenance organisation serving an urban, minority population	4	Cancer screening	Usual care	N; Group 1: Patient reminders only; Group 2: Physician reminder only; Group 3: Patient and physician reminder	A brightly colour notice placed in medical record for women who had mammography due (Group 2 and 3)	Visit to the primary care doctor and completion of a mammogram in the study year; approximately 12 months	Electronic records	OR 1.33 (1.01,1.75)
Chambers 1989 [93], RCT, USA	One family practice centre with 28 healthcare providers	2	Cancer screening	Usual care	N; Date of the last mammogram ordered and entered into the database was displayed in the comments section of the encounter form for each visit. This information was printed as ‘last mammogram: date’, or, if no mammogram was on record in the encounter form database (i.e. none since 1984), the notation was listed as ‘last mammogram?’	Date of the last mammogram ordered and entered into the database was displayed in the comments section of the encounter form for each visit	Up to date with the American Cancer Society guidelines for mammography (at end of intervention, 6 months follow-up)	Physician recorded ordering of mammograms on a patient encounter form which is entered into a patient registration database	OR 1.40 (1.08,1.82
Chambers 1991 [94], cluster RCT, USA	Family Practice Center of the Department of Family Medicine at Thomas Jefferson University	3	Vaccination	Usual care	N; Reminders identifying patients as eligible for the vaccine were printed on the encounter form according to the assigned group of the patient's primary physician. These reminders were provided for appropriate patients at every visit during the 2-month study period until the physician responded by ordering the vaccine. When the billing record showed the procedure had been performed, the computer programme removed the reminder message from the encounter form	Reminders identifying patients as eligible for the vaccine were printed on the encounter form according to the assigned group of the patient's primary physician	Percentage who received influenza vaccine; post-intervention (2 months)	Patient chart review (computerised database), adherence to the Immunization Practices Advisory Committee recommendation	OR 1.80 (1.09,2.98)
Fisher 2013 [95], RCT, Singapore	Three wards within 1 hospital (Singapore)	2	Hand hygiene	Usual care	Y; Wireless monitoring system of hand hygiene with reminders and individual feedback	Wireless monitoring system of hand hygiene with reminders and feedback	Hand hygiene compliance on entry/exit of patient zone within 10-week period	Electronic hand hygiene monitoring system. Compliance was registered when hand hygiene occurred within preset times of entering (6 s) or exiting (1 min) a patient zone	2.5 mth (entry) SMD 0.17 (−0.09,0.44) 2.5 mth (exit) SMD 0.39 (0.12,0.66) 1.5 mth (entry): SMD: 0.62 (0.35,0.89) 1.5 mth (exit) SMD: 0.45 (0.19,0.72)
Goodfellow 2016 [96], cluster RCT, England	30 general practices in the East Midlands of England	2	Obesity management	Usual care	Y; Tailoring, training and educational resources for healthcare professionals (including a presentation, discussion and provision of the resources, e.g. patient booklets, body mass index charts, calories and portions leaflets, posters, information on referral pathways)	Posters for consulting rooms containing information on how to measure waist circumference were given as a visual reminder	Proportion of overweight or obese patients to whom the health professional had offered a weight loss intervention within the study period; 9-month follow-up	Data collection was blinded and used a standard electronic system that extracted data from the general practice electronic health records and, to minimise bias, all data were collected using full anonymisation using electronic data extraction queries suitable for the different types of general practice computer systems used in England	OR 0.88 (0.45,1.72)
King 2016 [97], RCT, USA	One surgical intensive care unit at a hospital in Miami	4	Hand hygiene	Usual care	N; Visitors to an intensive care unit were exposed to an olfactory prime – a clean citrus smell that was introduced to the environment through a commercially available aroma dispenser. Visitors to an intensive care unit were exposed to a photo of eyes prominently displayed above the gel dispenser. In half the sessions a photo utilising clearly, female eyes was used and in the other photo male eyes	Olfactory prime (clean citrus small via aroma dispenser); Visual prime (photo of male or female eyes displayed prominently above the gel dispenser)	Observed hand hygiene compliance, 12 sessions of 3-h observations over a 3-month period	Direct observation	OR 3.18 (1.82,5.55)
Lafata 2007 [98], cluster RCT, USA	15 primary care clinics	3	Bone density screening	Usual care	N; Group 1: Initial and 1-month follow-up patient mailings were sent to women receiving the intervention. A third was sent to only those whose result indicated a need for follow-up. Group 2: As for Group 1 + physician prompts	Physician prompt in the electronic medical record and a biweekly letter to physician	Percentage who had bone mineral density testing/screening; 12 months	Health record data	OR 3.34 (2.29,4.88)
Le Breton, 2016 [99], cluster RCT, France	144 GPs, who provided care for any reason to 20,778 patients eligible for colorectal cancer screening between June 2010 and November 2011	2	Cancer screening	Usual care	N; Three reminders were mailed to GPs at 4-month intervals	Reminders contained lists of patients who had not performed a scheduled faecal occult blood test (FOBT)	Patient adherence to FOBT screening within the 17-month study period	Patient review database from the main French statutory health insurance programme and local screening programme	OR 1.08 (0.95,1.23)
Lobach 1997 [100], RCT, USA	58 primary care providers (20 family physicians, 1 general internist, 2 physician’s assistants, 2 nurse practitioners, 33 residents), outpatient setting, diabetic patients	2	Diabetes management	Usual care	N; Computer-Assisted Management Protocol consistent with the diabetes guideline recommendations. Protocol was printed on the first page of the paper encounter form and provided the customised diabetes guideline recommendations based on practice standards and previously completed tests, and an area for handwritten updates by the clinician to capture data not previously stored in the medical record	The protocol generated a set of disease-specific care recommendations customised to an individual patient that advised the clinician regarding which studies/procedures should be done during the current visit and which studies/procedures were next due	Clinician compliance rate with regards to care guidelines for diabetes mellitus overall (number of recommendations completed/total number of recommendations due); 6-month study period	Medical chart audit & review of computer-generated lab test summaries	SMD 0.97 (0.75,1.19)
Martin-Madrazo 2012 [101], cluster RCT, Spain	198 healthcare workers within 11 primary healthcare centres	2	Hand hygiene	Usual care	Y; Multimodal strategy based on World Health Organization (WHO) – posters; education sessions, availability of alcohol-based hand rub (4 × 50-min teaching sessions)	Hydroalcoholic solutions were placed in each consultation office	Hand hygiene compliance level; 6-month follow-up	Direct observations.	OR 2.93 (1.18,7.29)
Munoz-Price 2014 [102], cross-over RCT, USA	40 anaesthesiology providers at one, 1500-bed teaching hospital in Florida	2	Hand hygiene	Minimal intervention: Wall-mounted hand sanitiser dispensers only	N; Intervention involved using a hand sanitiser dispenser on the anaesthesia machine in addition to the standard wall-mounted dispensers	Additional hand sanitiser dispenser on anaesthesia machine	Frequency of hand hygiene, defined as the number of hand hygiene events per hour of observation, within 30 days, the same subjects were evaluated again in the opposite allocation	Direct observation	SMD 0.44 (−0.19,1.07)
Rogers 1982 [103], RCT, USA	Physicians, 479 Northwestern University Clinic patients	2	Hypertension management	Usual care	N; A computer printout of a current computerised medical record system summary in addition to the traditional medical record	A computerised medical record system was developed to provide physicians with concise and current information on patient’s problems, to identify omissions in recording of observations and treatment recommendations, to show ordered procedures that were not carried out, to record deficiencies in medical reasoning, and to recommend corrective actions according to selected criteria	Hypertension renal function examination (done both years). Obesity: number of diets given of reviewed (done both years). Renal disease renal function examination (done both years); for 2-year period	Blind retrospective chart reviews by trained personnel using a standardised evaluation form. Measurement tool was developed by the research team	Hypertension renal function examination OR 1.54 (1.03,2.31) Renal disease renal function examination OR 1.89 (0.85,4.20) Obesity number of diets given or reviewed OR 2.01 (0.96,4.23)
Rossi 1997 [27], cluster RCT, USA	71 primary care providers within one general internal medicine clinic	2	Prescribing	Usual care	N; Reminder was attached to the medication refill forms that are given to providers at every patient visit	One-page guideline reminder placed in the patient chart by the clinic pharmacist. The reminder highlighted the prescription and offered alternative drugs and doses	Prescription change rate. The percentage changed from calcium channel blocker after 6 months	Patient chart review via computer system	OR 30.40 (4.08,226.35)
Schnoor 2010 [104], RCT, Germany	8 Local Clinical Centres (11 hospitals & 34 sentinel practices)	2	Prescribing	Usual care	Y; Audit & feedback, educational meetings with dissemination of guideline, reminders	GPs and physicians received a poster, a short-printed version and an electronic version of the guideline	Adherence to the guideline was analysed for the following variables: initial site of treatment, empiric initial antibiotic treatment and duration of antibiotic treatment. After a training period of 1 month, process of care after guideline implementation (1 April 2007 to 29 February 2008) was compared with the treatment before (1 September 2006 to 28 February 2007)	Data of the recruited cases were entered by the personal tutor in-time, electronically using a standardised electronic report form (case report form) in a central database	Antibiotic treatment in outpatients OR 1.27 (0.91,1.77) Duration of antibiotic treatment in inpatients OR 0.93 (0.65,1.32) Duration of antibiotic treatment in outpatients OR 2.11 (1.47,3.02) Antibiotic treatment in inpatients OR 1.70 (1.19,2.42) Initial site of treatment OR 1.75 (1.21,2.55)
Shojania 1998 [105], RCT, USA	396 physicians in one tertiary-care teaching hospital	2	Prescribing	Usual care	N; A computerised guidelines screen appeared whenever a clinician in the intervention group initiated an order for intravenous vancomycin. Another guidelines screen is displayed after 72 h of therapy asking providers their indication for continuing vancomycin use	Showing computerised guidelines for vancomycin ordering at the time of initial vancomycin ordering and after 72 h of therapy	Number of vancomycin orders and duration of vancomycin therapy prescribed by providers; 9-month period	Vancomycin orders were obtained from computer log, monthly utilisation of vancomycin in the hospital was obtained from the pharmacy system.	Total number of vancomycin orders SMD 0.22 (0.02,0.41) Vancomycin days per physician SMD 0.23 (0.02,0.44)
Thompson 2008 [106], cluster RCT, England	19 acute mental health units in 4 local mental health trusts (667 nurses/doctors)	2	Prescribing	Minimal intervention: Received guidelines on antipsychotic polypharmacy	Y; An educational/cognitive behavioural therapy workbook; an educational visit to consultants; a reminder system on medication charts	A medication chart reminder system was developed. Ward pharmacists applied removable reminder stickers to medication charts when participants were prescribed more than 1 antipsychotic	Antipsychotic polypharmacy prescribing rates for each unit (cluster); 5 months	Information was collected from patients’ medication charts using a 1-day cross-sectional survey of antipsychotic prescribing pre- and post-intervention	OR 1.05 (0.66,1.68)
Yeung 2011 [107], cluster RCT, Hong Kong	Six residential long-term care facilities (188 nursing staff)	2	Hand hygiene	Intervention: Attended basic life support workshop (not hand hygiene)	Y; Education sessions, feedback, reminders	Pocket-sized containers of antiseptic hand rub were provided and kept close to clinicians.	Adherence to hand hygiene; 2-week intervention period followed by 7-month post-intervention	Direct observation	OR 1.17 (0.72,1.90)
Norms and messenger nudges									
Cranney 2008 [108], cluster RCT, Canada	119 primary care practices (174 clinicians)	2	Osteoporosis management	Usual care	N; Letter to patient and physician at 2 weeks and 2 months post-fracture	A personalised letter notified the physician that their patient had a recent wrist fracture and highlighted that wrist fractures can be associated with osteoporosis, and that assessment for osteoporosis treatment is recommended for women with wrist fractures	Proportion of women who reported they were started on osteoporosis treatment (i.e. bisphosphonates, raloxifene, hormone therapy or teriparatide) within 6 months of fracture, 6 months post-fracture	Self-report via telephone survey	OR 3.29 (1.65,6.55)
Engers 2005 [109], cluster RCT, Denmark	67 eligible GPs, 531 patients with nonspecific low back pain	2	Low back pain management	Usual care	Y; Two-hour workshop; distribution of a half-page patient education card; the guideline for occupational physicians; 2 scientific articles concerning GP management of nonspecific low back pain; and a collaboration tool to facilitate greater agreement with physical, exercise, and manual therapists on the management of nonspecific low back pain	In addition to the workshop, GPs received printed materials including patient education, a copy of the guidelines, scientific articles (educational material) and a collaboration tool	Number of referrals to a therapist (physical, exercise, or manual therapist) within 8-month study period	GPs completed self-registration forms post consultation; patient questionnaire completed immediately after the consultation	OR 5.17 (1.73,15.39)
Feldstein 2006 [110], RCT, USA	Nonprofit, group-model health maintenance organisation in the Pacific Northwest with about 454,000 members, 35% of whom were aged 50 and older and more than 90% of whom had a prescription drug benefit. 15 primary care clinics and 159 primary care providers (range 1–3 patients per provider)	3	Osteoporosis management	Usual care	Y; Group 1: electronic medical record message about participant risk of osteoporosis and distribution of educational materials Group 2: As for Group 1 + patient-directed component	Primary care providers received patient-specific electronic medical record in-basket messages for their enrolled patients from the chairman of the osteoporosis quality improvement committee	The primary outcome was the proportion of the study population who received a pharmacological treatment or a bone mineral density measurement within 6 months after the intervention	Identified electronically from the outpatient pharmacy system of data from the referral site (on bone mineral density measurement)	OR 15.93 (2.13,118.93)
Majumdar 2008 [111], RCT, Canada	Two largest emergency departments and 2 largest fracture clinics in Capital Health (Edmonton, Alberta) (266 primary care physicians)	2	Osteoporosis management	Minimal intervention: Mailed osteoporosis guidelines	Y; Distribution of guidelines endorsed by local leaders; physician reminder; patient telephone counselling	Evidence-based treatment guidelines, representing an actionable summary of available osteoporosis guidelines and having endorsement from 5 local opinion leaders, were sent to these physicians	Starting treatment with a bisphosphonate within 6 months after the fracture, 6 months post-fracture	Patient self-report, confirmed through dispensing records at local pharmacies	OR 1.46 (0.70,3.08)
Mertz 2010 [112], cluster RCT, Canada	30 adult hospital wards in 3 acute care sites	2	Hand hygiene	Minimal intervention: Alcohol-based gel dispensers were installed outside all patient rooms with at least 1 hand wash sink in each room	Y; Installation of gel dispensers as per control group + performance feedback, educational meeting & resources	Clinical managers were asked to develop a target adherence level. Meetings were held biweekly to provide unit-specific feedback. The adherence rates were shown on a large whiteboard both graphically and numerically. After 6 months, a comparison with the rates of other intervention units was provided	Rates of hand hygiene adherence, evaluated at unit level, assessed weekly for 1-year intervention period	Direct observations	OR 1.25 (0.90,1.74)
Shah 2014 [113], cluster RCT, Canada	80 primary care practices 1592 patients at high risk for cardiovascular disease were selected	2	Prescribing	Minimal intervention: Control providers received the Canadian Diabetes Association newsletter, which included the revised guidelines for cardiovascular disease screening	N; Printed educational materials (1 toolkit including guidelines summary, laminated card with risk assessment algorithm, self-assessment tool & risk reduction strategies)	Letter from the Chair of the practice guidelines Dissemination and Implementation Committee, with guideline summary	Prescription for statin; 10 months	Trained registered nurse undertook patient chart review	OR 0.76 (0.42,1.37)
Salience/affect nudges									
Dey 2004 [114], cluster RCT, England	24 General Practices with 2187 eligible patients	2	Test ordering	Usual care	Y; Educational outreach visit; guidelines (educational material); poster of guidelines; referral forms with guidelines; access to fast-track physiotherapy and a back clinic) General Practitioners (GPs) were sent a letter offering them a visit from the guideline team, followed by a telephone call to the practice manager to arrange an appointment with the GP in their practice. At least 2 members of the guideline team attended each visit. Members of the guideline team facilitated a structured interactive discussion with the GP	Face-to-face meeting included structured interactive discussion with the GP, which was based on the ‘elaboration likelihood model of persuasion’. This discussion was used to: raise awareness of the guidelines, adapt to the local context; emphasise the key messages in the guidelines; identify potential barriers to implementation; and suggest strategies for overcoming the barriers identified	The rate of referral for lumbar spine X-rays; 8 months	GPs were asked to log every patient presenting to them with acute low back pain: the practice was reimbursed £1 for each patient identified. A research assistant screened the records of these patients to confirm eligibility and to extract data on patient characteristics and clinical management during the 3-month period following first consultation	OR 0.89 (0.60,1.32)
Grant 2011 [115], RCT, USA	One hospital; with all 66 soap & gel dispensers randomly allocated to 1 of 3 signs	3	Hand hygiene	Minimal intervention: The control sign, which was developed by hospital managers, read, ‘Gel in, wash out’	N; Three different signs over period of 2 weeks	Personal consequences’ sign read ‘Hand hygiene prevents you from catching diseases’. The patient-consequences sign read, ‘Hand hygiene prevents patients from catching diseases’	Mean percentage of soap and gel used during 2-week periods before and after signs were introduced	Measured by blinded environmental services team	SMD 0.17 (−0.35,0.69)
Ince 2015 [116], RCT, England	13 community mental health teams (82 individuals)	2	Delivery of psychological interventions	Minimal intervention: Summary of guidelines for psychological interventions for schizophrenia	N; Alternative text of National Institute for Health and Clinical Excellence Guidelines guidance for schizophrenia	Summary of guidelines re-written. Text was amended to personalise the message, use of behaviourally specific language. Checklist & decision tree produced & provided	Overall intention to follow the recommendations measured by a Theory of Planned Behaviour Total Scale Intention Score, number of participants providing psychological interventions (delivered, received training, supervision), at 1-month follow-up	Self-report questionnaire	Intention to follow Theory of Planned Behaviour Total Scale Intention Score : SMD: 0.00 (−0.47,0.48) Received psychological training in last month OR 0.77 (0.16,3.76) Psychological interventions delivered OR 1.65 (0.58,4.66) Supervision for psychological interventions was used OR 1.69 (0.58,4.92)
Leslie 2012 [117], RCT, Canada	Unclear. There were *n* = 4264 patients randomised	3	Osteoporosis management	Usual care	Y; Group 1: Notification letter to primary care physician (reminder) about the patient’s fracture accompanied by educational material Group 2: As for Group 1 + patient-directed intervention (educational material and reminder)	A letter directed the physician to the provincial guidelines on bone mineral density testing and provided information on the management of osteoporosis. Additional information specific to the investigators research initiative was also provided. Enclosed with the letter were a requisition for a bone mineral density test and a flowchart showing the management of care	Combined end point of post-fracture bone mineral density testing or the start of medication for osteoporosis; 12 months post-fracture	Healthcare database information	OR 2.58 (2.17,3.07)
Priming (MPC: memorandum pocket card); default (TRF: test request form)									
Daucourt 2003 [118], cluster RCT, France	Six volunteer general hospitals and 1412 thyroid function tests ordered in 1306 patients	4 (2 included in current review (TRF [test request form] & MPC [memorandum pocket card])	Test ordering	Intervention: Physicians in all groups received guidelines and were invited to a local information meeting where guidelines were presented and discussed	N; Replacing previous order sheet with new TRF and providing small summary of recommendations on card	TRF makes ordering of inappropriate tests impossible based on format of the form MPC designed to be summary of guidelines that can be kept in pocket	Proportion of thyroid function test ordering in accordance with guidelines at 4 weeks after guideline implementation	Research Assistant completed data collection grid from patient medical files and test requests, or by speaking with the prescriber	OR 2.18 (1.16,4.10)
Priming, norms and messenger nudges									
Eccles 2001 [119], RCT, England	247 General Practices enrolled. Data was abstracted from 1693 patients’ records of 162 GPs in 48 practices	4 (2 × 2 factorial design)	Test ordering	Minimal intervention: Distribution of educational materials (guideline)	N; Group 1: Distribution of educational materials; audit and feedback (number of practice referrals compared with peers) Group 2: Distribution of educational materials; reminders (messages on X-ray results) Group 3: Distribution of educational materials; audit and feedback; reminders All interventions had a 12-month duration	Referral guidelines were posted to all GPs. Feedback contained the number of requests for lumbar spine and knee radiographs made by the whole practice compared with requests made by all GPs in the study was sent to GPs at start of intervention period and 6 months later. Educational messages were attached to the reports of every knee or lumbar spine radiograph requested during the 12-month intervention	The number of each radiograph (knee, lumbar, spine) requested per 1000 patients registered with every practice per year for 2 years; the second year was the intervention period	Records of radiology departments	Mean lumbar spine radiographs SMD 0.34 (0.05,0.63) Mean knee radiographs SMD 0.39 (0.09,0.68)
Majumdar 2007 [120], RCT, Canada	40 pharmacies (targeted sample size), patients with a self-reported diagnosis of heart failure or ischemic heart disease who was not taking a study medication	2	Prescribing	Minimal intervention: Physicians of the control subjects were faxed only their most recent medication profile	N; Five physicians were consistently identified as opinion leaders and worked with the investigators to develop the study’s evidence summaries	One-off fax of evidence summary to physician with the patient’s most recent medication profile	Improvement of prescribing for efficacious therapies in patients with a chronic cardiovascular disease within 6 months of the intervention	Patient-level medication profiles generated at each community pharmacy; outcomes measured by compliance with evidence-based prescribing recommendations	OR 1.46 (0.70,3.08)
McAlister, 2006 [121], RCT, Canada	Physicians at primary care practices, patients with established coronary artery disease	3	Prescribing	Minimal intervention: Physicians received a fax containing the coronary artery diagram for their patient	N; Opinion leader statement group: The opinion leader statements were imprinted with the name of the participating patient, addressed directly to the patient’s physician, signed by the local opinion leaders for that city, and faxed automatically by a software programme that was developed for this trial. Unsigned statement group: The unsigned statements were identical to the opinion leader statements in content and form but did not contain the opinion leaders’ signatures. The unsigned statements were faxed to physicians in the same manner as the opinion leader statements	Each physician received a fax containing objective evidence of the patient’s coronary artery disease (in the form of a coronary artery diagram) and either a signed or unsigned statement. These faxes were sent to physicians within a few days of the angiogram	Improved statin management, defined as initiation or increased dosage of a statin within the first 6 months after cardiac catheterisation	Medication outcomes were based on patient self-report (with cross-referencing to pharmacy records), and laboratory data and clinical outcomes were extracted from medical records	OR 1.31 (0.89,1.92)
Rodriguez 2015 [122], cluster RCT, Argentina	705 healthcare workers in 11 intensive care units at acute care hospitals	2	Hand hygiene	Usual care	Y; Educational resources, reminders, feedback, executive support	Every month, coordinators of intervened sites received results of the indicator (compliance with hand hygiene) and they showed them in the storyboard comparing it to the best performance in study (if the site complied with <70%) or to an international performance of 95% (if the site complied with 71% or more. Reminders placed at the entrance of patient’s rooms and in common areas	Adherence to hand hygiene based on the WHO survey tool; monthly for 9 months	Direct observation (covert)	OR 1.58 (1.09,2.29)
Schouten 2007 [123], cluster RCT, Netherlands	Six medium-large hospitals in southeast of the Netherlands	2	Prescribing	Not reported	Y; Audit & feedback; educational meetings with dissemination of guidelines	Consensus ‘critical-care pathways’ were distributed to all doctors as a laminated, pocket card; desktop and personal digital assistant versions were also distributed. Feedback on indicator performance at the hospital level was presented and provided in writing to all doctors treating hospital lower respiratory tract infections. Feedback reports included benchmarks at the hospital level (best practice) and presented key issues for improvement	A sum score was calculated that determined the sum score for guideline adherence for empirical antibiotic therapy; 2 years	All data were collected by concurrent chart review; trained research assistants made twice-weekly reviews of the charts of all patients who were admitted to the internal and respiratory medicine wards	OR 2.16 (0.75,6.23)
Taveras 2015 [124], cluster RCT, USA	Primary care practice paediatric clinicians, children with obesity	3	Obesity management	Usual care	Y; Modified electronic health record to deploy a computerised, point-of-care clinical decision support alert to paediatric clinicians at the time of a well-child visit for a child with a body mass index at the 95th percentile or greater. Clinicians were trained to use brief motivational interviewing to negotiate a follow-up weight management plan with the patient and their family. A comprehensive set of educational materials were developed for paediatric clinicians to provide to their patients	An alert containing links to growth charts, evidence-based childhood obesity screening and management guidelines, and a prepopulated standardised note template specific for obesity	Body mass index percentile documentation, Healthcare performance/quality of care (nutrition/physical activity counselling documentation); baseline and 1-year follow-up	Child’s electronic health record from well-child visits, and Healthcare Effectiveness Data and Information Set (health performance)	HEDIS Performance Measures BMI percentile documentation OR 1.49 (0.73,3.01) HEDIS Performance Measures nutrition PA counselling documentation OR 63.37 (3.81,1052.67)
Rahme 2005 [125], cluster RCT, Canada	GPs in eight small towns in Quebec, Montreal	3	Prescribing	Did not report	Y; Group 1: Workshop which discussed evidence-based management of patients with osteoarthritis Group 2: Decision trees to support decision-making Group 3: Combination of workshop and decision tree	Continuing medical education points and endorsement by medical bodies, delivered by peers (Groups 2 and 3)	Number of dispensed prescriptions for osteoarthritis from the Provincial Health Care fund database; 5 months pre-intervention and 5 months post-intervention (12 months)	Patient records	OR 1.52 (0.65,3.57)
Priming, salience, norms and messenger nudges									
Roux 2013 [126], RCT, Canada	Primary care physicians of an acute care hospital, 1446 patients aged 50 years or older with fragility fractures	3	Osteoporosis management	Usual care	Y; Group 1: Verbal and written information on osteoporosis to patient (patient-directed component) and letter with specific management plan sent to their treating physician (GP reminder). Patient reminders at 6 and 12 months. Reminder to physician if patient untreated at 6 months Group 2: As for Group 1 + blood tests and bone mineral density test ordered for patient and results sent to the physician (patient-mediated intervention). Patient reminders at 4, 8 and 12 months and physician reminders at 4 and 8 months if patient remained untreated	Verbal and written information on osteoporosis to patient and letter with specific management plan sent to their treating physician. Blood tests and bone mineral density test ordered for patient and results sent to the physician. Patients and physicians received reminder if patients remained untreated	Percentage change in treatment rates for osteoporosis; 1-year post-fracture	Delivery of osteoporosis medication was confirmed with the patient’s pharmacists	OR 3.05 (2.01,4.63)
Solomon 2001 [127], RCT; USA	17 internal medicine services within one academic medical centre in USA	2	Prescribing	Usual care	Y; Educational meetings with policy dissemination; 1 x face-to-face or telephone academic detailing session with clinician who wrote the order for the 2 unnecessary antibiotics being studied	Academic detailing, patterns of antibiotic utilisation and resistance patterns in the institution	Number of days that unnecessary antibiotics (levofloxacin or ceftazidime) were administered in intervention & control services; 18-week period	Computerised pharmacy records (validated in a sub-sample of patients against the manually completed medication administration records in patient chart)	SMD 1.54 (0.44,2.64)
Norms and messenger, salience and incentive nudges									
Robling 2002 [128], cluster RCT, Wales	39 general practices in South Glamorgan, Wales	4	Test ordering	Minimal intervention: single A4-sheet feedback on practice data	Y; Seminar workshop facilitated by academic GPs and researcher; videos; question and answer session	Continuing medical education point, feedback from experts, presentation of localised guidelines	Percentage concordant with local guidelines (MRI: medical resonance imaging requests); 11 months	Each MRI request was followed up, additional information assessed via follow-up interview with GPs	OR 0.59 (0.24,1.42)
Norms and messenger, priming and incentive nudges									
Solomon 2007 [129], cluster RCT, USA	828 primary care physicians within primary care clinics	4 (2 × 2 factorial design) (only relevant doctor arm described)	Prescribing	Usual care	Y; Educational resources that were used in a face to face educational session. Osteoporosis treatment algorithms, reminders flags and behavioural prescription packs were also provided	One hour of continuing medical education credit by Harvard Medical School were offered; reminder flags	Composite score consisting of either undergoing bone mineral density testing or initiation of medication for osteoporosis; 12 months	Patient Medicare and pharmacy claims data	OR 0.89 (0.74,1.06)
Norms and messenger, priming, salience and commitment nudges									
Stewardson 2016 [130], cluster RCT, Switzerland	Hospital ward	3	Hand hygiene	Intervention: Standard multimodal hand hygiene promotion activities, including monitoring and feedback, were done hospital wide throughout the study	Y; Group 1: Audit and feedback; goal setting; executive support Group 2: As for Group 1+ patient participation (educational materials, alcohol-based handrub)	Immediate verbal feedback and, where feasible, a card reporting individual hand hygiene compliance and individualised written advice for how to improve were provided. The card also illustrated the WHO Five Moments for Hand Hygiene and stated the institution-wide hand hygiene compliance goal (≥80%), with the signatures of the medical and nursing directors	Overall hand hygiene compliance of healthcare workers, at least once every 3 months during the baseline and intervention periods, and once every 4 months during the follow-up period	Direct observation during 20-min sessions	OR 1.10 (0.84,1.44)

Note: *RCT* randomised controlled trial, *USA* United States of America, *GP* general practitioner, *FOBT* faecal occult blood test, *WHO* World Health Organization, *MRI* medical resonance imaging

### Methodological quality of included studies

The risk of bias for each RCT as reported in the Cochrane reviews is presented in Table 3. Over half of the trials were judged as low risk for selection bias (random sequence generation (*n* = 33), allocation concealment (*n* = 28) and attrition bias (incomplete outcome data (*n* = 32)). A large number of trials were judged as unclear for selective reporting (*n* = 20) and protection against contamination (*n* = 14). In terms of blinding of outcome assessment, 21 trials were judged as having a low risk of bias (see Table 3). Overall, 29 studies (69%) met at least half of the criteria they were assessed against.

**Table 3:** Risk of bias assessments from individual trials extracted from published Cochrane reviews

Author name, year, study design, country	Random sequence generation	Allocation concealment	Protection against contamination	Baseline outcomes similar	Baseline characteristics similar	Blinding of outcome assessment	Incomplete outcome data	Selective reporting	Other bias
Baer 2016, cluster RCT, USA	Low risk	Unclear risk	Low risk	Not assessed	Not assessed	High risk	High risk	High risk	Unclear risk
Barnett 1983, RCT, USA	Unclear risk	Unclear risk	High risk	Unclear risk	Low risk	Unclear risk	Low risk	Unclear risk	Unclear risk
Burack 1996, RCT, USA	Unclear risk	Unclear risk	High risk	Unclear risk	Low risk	Low risk	Low risk	Unclear risk	Unclear risk
Chambers 1989, RCT, USA	Low risk	Unclear risk	High risk	Low risk	Low risk	Unclear risk	Unclear risk	Unclear risk	Unclear risk
Chambers 1991, cluster RCT, USA	Low risk	Unclear risk	Low risk	Unclear risk	Unclear risk	Unclear risk	Low risk	Unclear risk	Unclear risk
Cranney 2008, cluster RCT, Canada	Low risk	Low risk	Unclear risk	Low risk	Low risk	Unclear risk	Low risk	Unclear risk	Low risk
Daucourt 2003, cluster RCT, France	Low risk	Low risk	Not assessed	Unclear risk	Unclear risk	Unclear risk	Low risk	Low risk	Low risk
Dey, 2004, cluster RCT, England	Low risk	Low risk	Unclear risk	Unclear risk	Low risk	High risk	Low risk	Unclear risk	Low risk
Eccles, 2001, RCT, England	Low risk	Low risk	Low risk	High risk	Unclear risk	Low risk	Low risk	Unclear risk	Unclear risk
Engers, 2005, cluster RCT, Denmark	Low risk	High risk	Unclear risk	Unclear risk	Unclear risk	High risk	Low risk	Unclear risk	High risk
Feldstein 2006, RCT, USA	Low risk	Low risk	Unclear risk	Unclear risk	Low risk	Low risk	Low risk	Unclear risk	Unclear risk
Fisher 2013, RCT, Singapore	Low risk	Low risk	Unclear risk	Low risk	High risk	High risk	Low risk	Low risk	Low risk
Goodfellow 2016, cluster RCT, England	Low risk	Low risk	Low risk	Low risk	Unclear risk	Low risk	Unclear risk	Low risk	Not assessed
Grant 2011, RCT, USA	Unclear risk	Low risk	Low risk	Low risk	High risk	Low risk	Low risk	Low risk	Low risk
Ince 2015, RCT, England	Low risk	Low risk	Not assessed	Not assessed	Not assessed	Low risk	Unclear risk	Low risk	Low risk
King 2016, RCT, USA	Low risk	Low risk	Unclear risk	Low risk	Low risk	High risk	Low risk	Low risk	Unclear risk
Lafata 2007, cluster RCT, USA	High risk	Unclear risk	Unclear risk	Low risk	Unclear risk	High risk	High risk	Unclear risk	Low risk
Le Breton, 2016, cluster RCT, France	Low risk	Low risk	Low risk	Low risk	High risk	Low risk	Low risk	Low risk	Unclear risk
Leslie 2012; RCT; Canada	Low risk	Low risk	Unclear risk	Low risk	Low risk	Low risk	Low risk	Low risk	Low risk
Lobach, 1997, RCT, USA	Low risk	Low risk	Low risk	Low risk	Low risk	Low risk	Low risk	Unclear risk	Unclear risk
Majumdar 2007, RCT, Canada	Low risk	Low risk	High risk	Unclear risk	Unclear risk	Low risk	Low risk	Unclear risk	Low risk
Majumdar 2008, RCT, Canada	Low risk	Low risk	High risk	Unclear risk	Unclear risk	Low risk	Low risk	Unclear risk	Low risk
Martin Madrazo 2012, cluster RCT, Spain	Low risk	Low risk	Low risk	Low risk	High risk	Low risk	Low risk	Low risk	High risk
McAlister, 2006, RCT, Canada	Low risk	Low risk	Low risk	Low risk	Low risk	Low risk	Low risk	Low risk	Low risk
Mertz 2010, cluster RCT, Canada	Low risk	Low risk	High risk	Low risk	High risk	Unclear risk	Low risk	Low risk	High risk
Munoz-Price 2014, cross-over RCT, USA	Low risk	Low risk	High risk	Unclear risk	Low risk	High risk	Low risk	Low risk	Low risk
Rahme 2005, cluster RCT, Canada	Unclear risk	Unclear risk	Low risk	Unclear risk	Low risk	Low risk	Low risk	Unclear risk	Unclear risk
Robling 2002, cluster RCT, England	High risk	Unclear risk	Unclear risk	Unclear risk	Unclear risk	Low risk	Unclear risk	Unclear risk	Unclear risk
Rodriguez 2015, cluster RCT, Argentina	Low risk	Unclear risk	Unclear risk	Low risk	Unclear risk	Unclear risk	Low risk	Low risk	Low risk
Rogers 1982, RCT, USA	Unclear risk	Unclear risk	Unclear risk	Unclear risk	Unclear risk	Low risk	High risk	High risk	Not assessed
Rossi 1997, cluster RCT, USA	Low risk	Low risk	Low risk	Unclear risk	High risk	Low risk	Unclear risk	Unclear risk	Unclear risk
Roux, 2013, RCT, Canada	Low risk	Low risk	Unclear risk	Low risk	High risk	High risk	Low risk	Unclear risk	Unclear risk
Schnoor 2010, RCT, Germany	Low risk	Low risk	Low risk	High risk	High risk	Unclear risk	Unclear risk	Unclear risk	High risk
Schouten 2007, cluster RCT, Netherlands	Low risk	Low risk	Low risk	Low risk	Low risk	Unclear risk	Low risk	Unclear risk	Low risk
Shah 2014, cluster RCT, Canada	Low risk	Low risk	Not assessed	Low risk	High risk	Low risk	Low risk	Unclear risk	Unclear risk
Shojania 1998, RCT, USA	Unclear risk	High risk	High risk	Unclear risk	Low risk	High risk	Unclear risk	Low risk	Low risk
Solomon 2007, cluster RCT, USA	Low risk	Unclear risk	Low risk	Unclear risk	Low risk	Low risk	Low risk	Low risk	Unclear risk
Solomon 2001, RCT, USA	Low risk	Unclear risk	Low risk	Unclear risk	Low risk	Low risk	Low risk	Low risk	Unclear risk
Stewardson 2016, cluster RCT, Switzerland	Low risk	Low risk	Unclear risk	Low risk	Unclear risk	High risk	Low risk	Low risk	Low risk
Taveras 2015, cluster RCT, USA	Low risk	Low risk	Low risk	Low risk	Unclear risk	Low risk	Low risk	High risk	Not assessed
Thompson 2008, cluster RCT, England	Low risk	Low risk	Not assessed	Not assessed	Not assessed	Unclear risk	Low risk	Low risk	Unclear risk
Yeung 2011, cluster RCT, Hong Kong	Unclear risk	Low risk	Unclear risk	Low risk	High risk	High risk	Low risk	Low risk	High risk

Risk of bias was assessed for the primary implementation outcome where specified

*Note*: Not assessed refers to where the risk of bias criteria were not assessed in the Cochrane review in which they were identified

### Application of nudge strategies to improve implementation

All nudge strategies were used in at least one trial to influence healthcare provider adherence to guideline recommendations. Twenty-two studies across 30 outcomes incorporated nudge strategies as part of a multicomponent intervention, while the remainder examined the impact of nudge strategies in isolation (*n* = 20). Ten studies applied two nudge strategies and three applied three or more nudge strategies. The frequency to which each strategy was used and specific examples of their application are shown in Table 4.

**Table 4:** Summary of application of nudge strategies in 42 randomised controlled trials included in this study

Nudge strategy	Application in RCTs included in this review	Number (*n*, %)
Priming nudge	Stickers displayed at point of care, displaying problematic scans to primary care providers when high risk patients presents, treatment reminders/flags on online records, visual prime (picture/smells) to prompt targeted behaviour, reminder posters in display area, story board with priority problems and endorsement visually displayed, availability of resources to prime targeted behaviour (pocket hand rub, ball pen, posters), pocket-sized cards/laminated messages, mailing of questions regarding targeted behaviour to prime thinking of action, point-of-care prompts to assess/screen, reminders to measure/assess in electronic medical records at point of care	29, 69%
Norms and messenger nudge	Sending guidelines by email/mail endorsed by a reputable organisation (e.g. chair, president or governing organisation), presenting information on performance relative to other providers and units in the area, letters signed by opinion leaders, resources endorsed by directors of the unit	17, 40%
Salience/affect/affect nudge	Presenting vignettes with relevant patients’ cases to physicians, personal consequences signs	8, 19%
Default nudge	Restricting options where not relevant to a particular patient/case (shading of boxes)	1, 2.4%
Commitment/ego nudge	Providers to publicly declare their commitment/ ego to reducing inappropriate implementation behaviour/conducting implementation behaviour, displaying participation in improvement initiatives publicly	1, 2.4%
Incentives nudge	Provision of continuing medical education points, certificates	3, 7.0%

#### Priming nudge

The most commonly used nudge strategy was priming, which was included in 69% (*n* = 29) of trials, across 41 outcomes. The impact of this nudge strategy was assessed on a range of implementation outcomes including adherence to antibiotic prescribing guidelines [104, 105, 123], prescribing rates of medication and test ordering of various conditions [27, 92, 93, 98, 99, 103, 106, 118, 119, 121, 129, 131], vaccinations [94], provision of care according to guidelines [90, 91, 96, 100, 103, 124, 126], and adherence to hand hygiene guidelines [95, 97, 101, 102, 107, 122, 130]. Priming nudges were also applied in various clinical settings including hospitals [95, 102, 104, 118, 122, 130], primary care practices [27, 91, 93, 94, 96, 99–101, 103, 119, 121, 124], mental health units [106] and community-based long-term care facilities [107].

#### Norms and messenger nudge

Norms and messenger nudge were the second most commonly used nudge strategy, included in 40% of studies (*n* = 17) across 19 outcomes. The impact of this strategy on a number of implementation outcomes including appropriate prescribing of medication [108, 111, 113, 125, 127, 132], and test ordering for various conditions [110, 119], antibiotic prescribing [123, 127], provision of preventive/lifestyle care according to guidelines [109, 124] and adherence to hand hygiene guidelines [112, 122, 130] was assessed. Interventions were undertaken in various clinical settings including hospitals [111, 112, 122, 127, 130], primary care practices [108–110, 113, 119, 121, 133] and pharmacies [131].

#### Salience and affect nudge

This was the third most frequently used nudge strategy, utilised in 19% of the studies reviewed (*n* = 8), across 11 outcomes. This was undertaken in interventions in hospitals [115, 127], primary care practices [114, 126] and community mental health teams [116] to improve test ordering (i.e. screening for bone mineral density) [114, 117], hand hygiene [115, 130], provision of care according to various guidelines (i.e. mental health) [116, 126, 128] and antibiotic prescribing [127].

#### Incentive, commitment/ego and default nudge

Less than 10% of studies incorporated either incentive (*n* = 3), commitment/ego (*n* = 1) or default (*n* = 1) nudge strategies as part of their interventions. The one study that used a commitment/ego nudge was conducted in hospitals to increase adherence to hand hygiene where clinicians publicly declared their commitment to either reduce inappropriate behaviour or increase recommended behaviour [130]. A default nudge was used in one study conducted in hospitals where test ordering options related to thyroid function not relevant to patients were shaded out based on ordering forms and incentive strategies [118]. The incentive strategy primarily included provision of certificates and professional development points [125, 128, 129] to primary care physicians to increase appropriate prescribing or test ordering.

### Impact of nudge strategies on healthcare provider behaviour

**Fig. 2:**
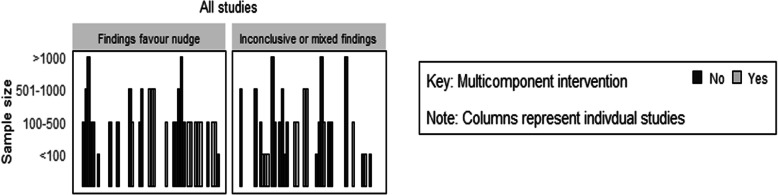
Harvest plot of estimated effect estimates for all include studies

Of the 57 outcomes assessed, 49 (86%) had an estimated effect on clinician behaviour in the hypothesised direction, of which 30 (53%) did not contain the null value (see Table 5). Figure 2 shows the distribution of studies by whether they had an estimated effect on the outcome. The median standardised mean difference across all continuous outcomes was 0.39 (IQ1 = 0.22, IQ3 = 0.45). For dichotomous outcomes, the median OR across all outcomes was 1.62 (IQ1 = 1.13, IQ3 = 2.76).

**Table 5:** A summary of the number and percentage of outcomes reporting an estimated effect in support of the intervention by number of nudge strategies and intervention type

Strategy	Number of studies	Number of outcomes	Number (%) of estimated effects in direction of hypothesised effect^a^	Number (%) estimated effects in direction of hypothesised effect and significant^a^
All studies	42	57	49 (86)	30 (53)
Nudge as part of multicomponent intervention	22	30	24 (80)	15 (50)
Nudge only intervention	20	27	25 (93)	15 (56)
One nudge strategy included	29	42	36 (86)	23 (55)
More than one nudge strategy included	13	15	13 (87)	7 (47)
Type of nudge strategies:				
Priming	29	41	37 (90)	24 (59)
Norms and messenger	17	19	16 (84)	9 (47)
Salience/ affect/affect	8	11	8 (73)	3 (27)
Incentive	3	3	1 (33)	0 (0)
Default	1	1	1 (100)	1 (100)
Commitment/ego	1	1	1 (100)	0 (0)

*Note*: ^a^There are several studies that report on more than one outcome and thus are represented more than once in this result

When the nudge strategy was included as part of a multicomponent intervention, 24 out of 30 (80%) had a calculated effect estimate that was in the hypothesised direction, of which 15 (50%) did not contain the null. Comparatively, 20 studies across 27 outcomes tested a nudge only intervention where 25 of these outcomes (93%) were in the hypothesised direction and 15 (56%) did not contain the null (see Table 5, Fig. 1).

**Fig. 3:**
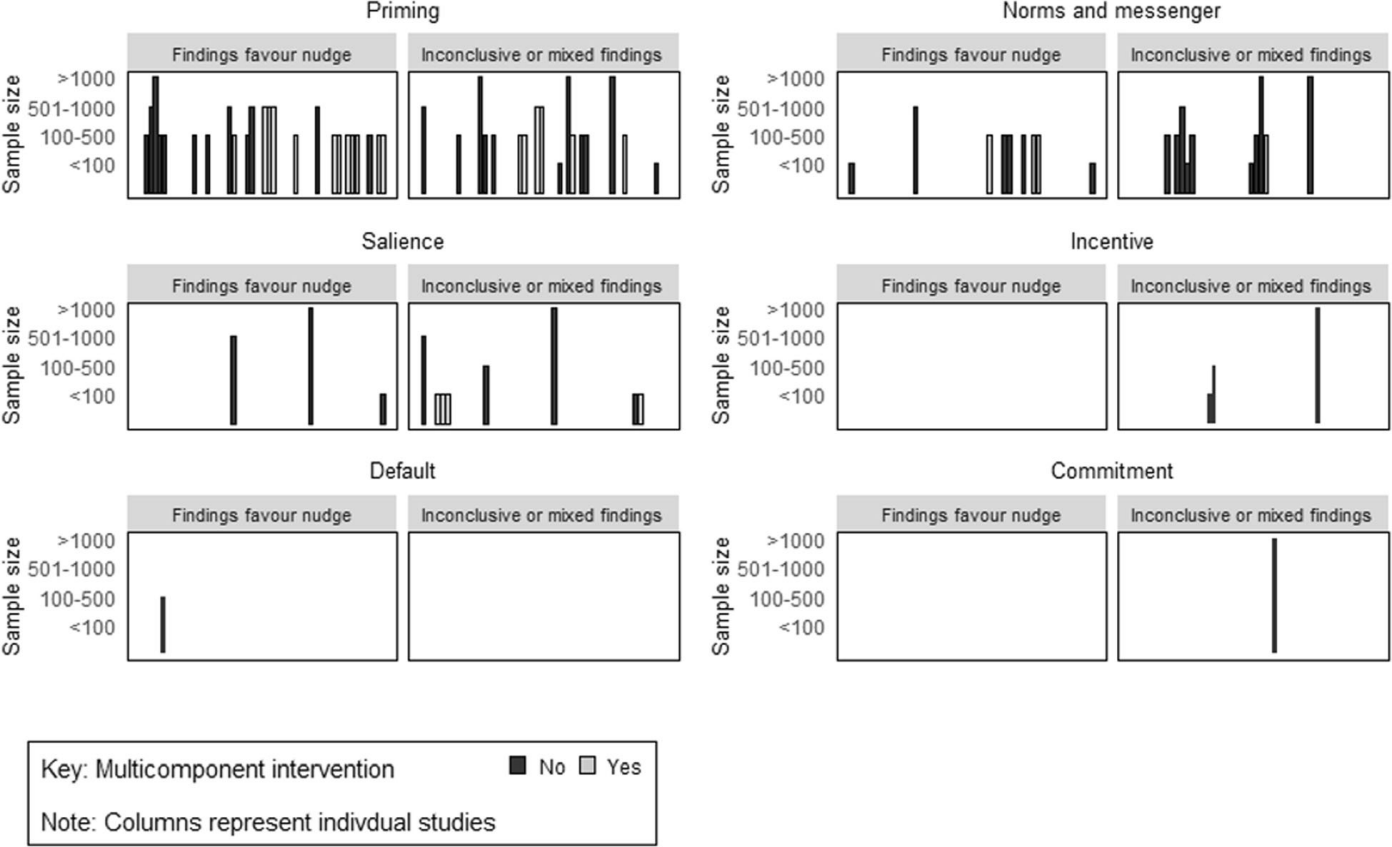
Harvest plot of estimated effect estimates by nudge classification  and whether studies were multi or single component studies

Twenty-nine studies across 42 outcomes included only one type of nudge strategy as part of their intervention packages. Of this, 36 (86%) were in the hypothesised direction, of which 23 (55%) did not contain the null. Comparatively, 13 studies across 15 outcomes employed intervention packages that included more than one nudge strategy. Of these, 13 of the 15 outcomes (87%) were in the hypothesised direction and 7 (47%) did not contain the null. See Table 5 and Fig. 3 for the number and percentage of outcomes by nudge strategies. Interventions that included a priming nudge showed the most promise, with 37 out of the 41 outcomes (90%) in the hypothesised direction.

The most consistent evidence of effect was observed for behaviours such as handwashing (all outcomes in the hypothesised direction, with 70% containing the null) [95, 97, 101, 102, 107, 112, 115, 122, 130], and test ordering and prescribing for management of osteoporosis (all outcomes in the hypothesised direction, with 80% containing the null) [108, 110, 111, 117, 126]. The effects were least consistent for obesity management (two out of four in the hypothesised direction, with 25% containing the null) [90, 96, 124].

## Discussion

This review of 42 RCTs included within eligible Cochrane systematic reviews found that a variety of nudge strategies have been employed in trials of clinical practice change interventions. For the majority of outcomes assessed (49/57), the effects were in the hypothesised beneficial direction. Additionally, a median effect size of 0.39 (IQ1 = 0.22, IQ3 = 0.45) for continuous and OR 1.62 ( 95% CI = 1.13, = 2.76) for dichotomous outcomes were calculated. These effects are comparable to other systematic reviews that have assessed the impact of a diverse range of implementation strategies on provider adherence to clinical guidelines [134, 135] and support the continued application of nudge interventions in practice. These comparable effects highlight the need to better consider and account for the potential impact of nudge strategies in the evaluation of complex implementation interventions, as these strategies are often overlooked.

Additionally, using vote-counting approaches, our study found that the effects of interventions were not related to the number of nudge strategies employed, or whether nudge strategies were part of a broader package of implementation support. While the evidence around this is mixed, these findings are consistent with a previous review of 25 reviews of implementation strategies, which found no compelling evidence that multicomponent strategies are more effective than single interventions [136]. As our findings relied on indirect comparisons, future studies testing the effects of nudge strategies using staggered or factorial trial designs are needed to better quantify the impact of individual nudge strategies by itself and in combination with other strategies.

Priming nudges were the most frequently evaluated, while few studies assessed the impact of incentive, commitment and ego nudges. While there were variable effects depending on outcomes, the most consistent effect was observed for handwashing, and test ordering and prescribing for osteoporosis management. It is possible that behaviours such as handwashing are more likely to be habitual, where practices are standardised and relatively simple to implement [137], and thus may respond to lower intensity stimuli. The outcomes measured for these behaviours are also likely to be more proximal and directly related to the intervention. These findings highlight the need to carefully consider the target behaviour and outcomes assessed when developing nudge interventions. Further, grounding the design and evaluation of nudge interventions in theory is needed to increase understanding of the context, type of behaviours and for whom nudge interventions may be effective for. Critically, future examination of the impact of nudge interventions according to different sociocultural characteristics (i.e. ethnicity, age, socioeconomic status) is needed to ensure such interventions do not exacerbate health inequalities.

Collectively, findings from this review highlight the positive effects of nudge interventions more broadly and support its application in clinical practice for some behaviours. It also identifies potential areas for development and testing of nudge strategies that have not been well studied such as for commitment nudges, where promising intervention effects have been reported [14]. For healthcare administrators, there is significant opportunity to embed priming, salience and default nudges within existing electronic systems or existing quality improvement tools such as reminders and audit and feedback. For example, scans or test results can be programmed to automatically pop up on electronic health records when a high-risk patient presents for care to facilitate follow-up. Additionally, electronic systems can be programmed to default to a more efficient treatment option where available (i.e. prescribing generic over brand name medications).

Where there is available infrastructure, embedding ‘nudge units’ similar to that described by Patel et al., within health services provides an innovative way of improving quality of care, while systematically developing and testing the impact of nudge interventions [138]. These units involve collaboration between health system administrators and leaders, front-line staff and researchers. Such collaborations enable the co-development of nudge solutions to address identified areas of suboptimal care. The unit is also then involved in subsequent implementation, evaluation and translation of the strategy, if shown to be effective [138]. The embedding of such units within clinical health services is similar to that previously described in public health practice [139] and provides significant opportunity to understand how nudge strategies can be best applied to improve clinical practice. Further, participation in innovative platforms such as the audit and feedback meta-lab [140], which involves collaboration between healthcare organisations and researchers, can provide an opportunity to further undertake head-to-head comparisons of nudge strategies applied within audit and feedback interventions.

Despite its promise, the application of nudge strategies into practice will firstly need to carefully consider issues around ethics and personal choice. As nudge interventions are inherently designed to influence the automatic systems, these attempts to change behaviour may be seen as challenging the role responsibility of clinicians and perceived as manipulative. Engaging clinicians early on with designing and implementing interventions is crucial to ensure the issues surrounding consent and freedom of choice are given due attention [12, 141].

Findings from this review should be considered in the context of a number of limitations. As there is no general consensus on the kinds of behavioural interventions that are classified as nudge, we used a pragmatic and systematic approach where we searched trials included in recently published Cochrane Reviews. This approach was selected as we sought to identify and characterise nudge implementation strategies across a range of medical disciplines and health conditions. As such, our approach undoubtedly failed to capture all eligible published trials, and findings described here are reflective of RCTs included in the published reviews. Future attempts to develop a more targeted electronic search strategy are likely to result in a more comprehensive review. The heterogeneity of outcomes, strategies and targeted guidelines precluded the use of meta-analysis to describe effects. We used non-meta-analytic methods of synthesis including providing a summary of overall effect estimate and vote-counting approaches. Vote-counting approaches are limited in its ability to examine intervention effectiveness as it is unable to account for differential weights in each study based on sample size. Further, seven studies had more than one primary outcome and were included more than once in the vote-counting procedure, which may have given more weight to such studies. While we attempted to extract risk of bias assessments for implementation outcomes, not all reviews specified this. As such, it is possible that the risk of bias assessment for these studies may be related to other non-implementation outcomes.

While such limitations exist, this review included 42 RCTs which provides a broad understanding of how nudge strategies have been applied to improve clinical practice and opportunities to further develop this important area of research. We used the Mindspace framework to provide a broad overview of the types of nudge intervention. To provide more insight into how nudge interventions can change behaviour, future reviews should consider using taxonomies such as the behaviour change techniques to classify these interventions by its psychological targets [142], or mapping to existing implementation taxonomies such as the Expert Recommendations for Implementing Change (ERIC) or EPOC taxonomy [143]. Our review also primarily focused on assessing the impact of the interventions on fidelity outcomes. Future reviews should consider assessing a broader range of implementation outcomes as specified by Proctor et al [144].

## Conclusions

### Main conclusion

This study is the first of its kind and offers new information for policy makers, practitioners and quality improvement agencies to support the application of nudge strategies to improve provision of clinical care. The review provides an overview on how such strategies have been applied and some evidence demonstrating the positive effects of nudge strategies. While more definitive research is needed, the results of this review suggest that nudges could be an effective tool to improve implementation of some clinical guidelines.

### Future research

In addition to primary research exploring the effects of nudge interventions on clinical practice, there is considerable need to develop standard terminology that is applied consistently to describe these strategies as well as detailed guidance on describing such interventions. Currently, the diversity and inconsistency in the terminology represents a real barrier to synthesising, applying and advancing the field. Such work could be considered a priority in the field of implementation science given the considerable research investment and abundance of randomised trials assessing the effects of strategies target rational clinical decision-making.

## Supplementary information

**Additional file 1.** Estimated effect estimates for continuous data used to generate results

**Additional file 2.** Estimate effect estimates for dichotomous data used to generate results

## Data Availability

The datasets supporting the conclusions of this article are included within the article (and its additional files).

## Abbreviations

RCT: Randomised controlled trial
MINDSPACE: Messenger, Incentives, Norms, Defaults, Salience, Priming, Affect, Commitments, Ego
OR: Odds ratios
EPOC: Cochrane Effective Practice and Organisation of Care
BCTT: Behaviour Change Technique Taxonomy
ERIC: Expert Recommendations for Implementing Change
